# Adverse Drug Reaction to Linezolid in Drug-Resistant Tuberculosis: A Systematic Review

**DOI:** 10.3390/medsci14010003

**Published:** 2025-12-22

**Authors:** Emy Oktaviani, Kusnandar Anggadiredja, Lia Amalia

**Affiliations:** Department of Pharmacology and Clinical Pharmacy, School of Pharmacy, Bandung Institute of Technology, Bandung 40132, Indonesia; 30722302@mahasiswa.itb.ac.id (E.O.); kusnandar_a@itb.ac.id (K.A.)

**Keywords:** linezolid, hematological, neurotoxicity, adverse drug reactions

## Abstract

**Background/Objectives**: The use of linezolid in drug-resistant tuberculosis has shown good effectiveness but has a high risk of adverse drug reactions (ADRs). Linezolid-related ADRs have been widely reported and may affect their therapeutic effect. This systematic review aimed to describe linezolid-related ADRs in drug-resistant tuberculosis. **Methods**: This literature review was conducted on PubMed, Scopus, ProQuest, and Sage without year limitation, up to June 2023. Study quality was assessed using the JBI checklist to evaluate method quality and risk of bias in the included articles. Inclusion criteria included studies assessing linezolid-correlated ADRs in drug-resistant tuberculosis patients with individual regimens, having access to the full text, and using the English or Indonesian language. Potential reporting bias was minimized by comprehensive database search and duplicate screening. **Results:** Initially, we identified 650 potential studies. Upon further assessment for relevance and eligibility, seven articles were selected for analysis. From seven articles, it was shown that all articles were reporting about linezolid-correlated ADRs. The three main ADRs are hematologic toxicity, peripheral neuropathy, and optic neuritis. In addition, gastrointestinal disorder and hyperlactatemia are reported as ADRs too. Varied doses of linezolid were used in the seven articles; they range from 300 mg to 1200 mg, with 600 mg/twice daily and 1200 mg/day being dominant. **Conclusions**: Linezolid-associated ADRs are dose- and duration-dependent. Hematological toxicity most commonly occurs at the beginning of treatment, while peripheral neuropathy and optic neuritis appear after long-term use. Therefore, intensive monitoring and therapeutic drug monitoring are essential to ensure the safety of linezolid therapy.

## 1. Introduction

Drug-resistant tuberculosis (TB) remains one of the most prevalent infectious diseases worldwide. Nonetheless, more than 10 million people continue to fall ill with TB every year, and more than 1 million die from the disease, making it the world’s leading cause of death from a single infectious agent. Globally, in 2024, an estimated 10.7 million people fell ill with TB as incident cases, and 1.23 million died from the disease. The TB incidence rate was 131 cases per population of 100,000 per year, and the case fatality rate was 11.5%. Most people who develop TB disease each year are in 30 high TB-burden countries, and Indonesia (10%) is among the top 8 countries with a high TB burden. Indonesia is also one of the global TB watchlist countries, characterized by high TB incidence and substantial gaps between estimated TB cases and notifications, with Indonesia included among the 10 countries with the largest reporting gaps. Globally, from 2020 to 2024, there has been a declining trend in TB cases. The net reduction from 2015 to 2024 was 12%, far from the End TB Strategy milestone of a 50% reduction by 2025. During the World Health Assembly in May 2025, Indonesia was reassigned from the South-East Asia Region to the Western Pacific Region, and for trend analyses, Indonesia was included in the Western Pacific Region throughout the report [1].

Drug-resistant TB continues to be a public health threat. TB that is resistant to rifampicin and isoniazid is defined as multidrug-resistant TB (MDR-TB), while TB that is resistant only to rifampicin is defined as rifampicin-resistant (RR-TB). Both MDR-TB and RR-TB require treatment with second-line drugs. Globally, the estimated annual number of people who develop MDR-TB has been declining since 2015. The estimated number in 2024 was 390,000. In 2024, the estimated proportion of new TB cases that were MDR-TB was 3.2%, a decrease from 4.7% in 2015. The estimated proportion of MDR-TB among people with a previous history of TB treatment was higher, at 16% in 2024. Indonesia is one of the 30 high MDR/RR-TB-burden countries. In 2024, Indonesia was among the 24 high MDR/RR-TB-burden countries that achieved at least 80% coverage for testing for rifampicin-resistant TB (RR-TB), reflecting substantial progress in diagnostic access [1].

The treatment success rate for drug-susceptible TB has remained high in recent years. Globally, it was 88% in 2023 and in 2022, an increase from 87% in 2021 and 86% in 2020. Meanwhile, the treatment success rate among people with MDR/RR-TB has shown considerable progress. In 2022, the treatment success rate reached 71%, up from 50% in 2020, 68% in 2021, and 64% in 2020 [1]. Based on Indonesia’s national TB data reported by the Ministry of Health, TB case notifications in 2024 reached 640,541 cases, reflecting a decline in incidence compared to 2023 and 2022. In 2024, the treatment success rate in Indonesia was 85% for drug-susceptible TB and 59% for MDR/RR-TB [2].

Since 2018, the WHO has recommended all-oral regimens for the treatment of MDR/RR-TB. The latest recommendations for the treatment of drug-resistant TB include three major categories of regimens. The first category is two 6-month all-oral regimens for people with MDR/RR-TB (with or without resistance to fluoroquinolones). The first regimen, referred to as BPaLM, comprises bedaquiline, pretomanid, linezolid, and moxifloxacin. The other regimen, referred to as BDLLfxC, comprises bedaquiline, delamanid, and linezolid, combined with levofloxacin or clofazimine, or both. Unlike BPaLM, the latter can be used in children and during pregnancy. The second category consists of several all-oral short regimens of 9 months for people with MDR/RR-TB who do not have any resistance to fluoroquinolones, and the last category is longer regimens of 18–20 months that may include an injectable drug (amikacin). All of these regimens have been used in Indonesia for MDR/RR-TB patients. Longer regimens (18–20 months) remain the most widely used [1,3].

Linezolid is one of the drugs used in the treatment of drug-resistant TB. Originally developed for infections caused by methicillin-resistant *Staphylococcus aureus* (MRSA) and vancomycin-resistant *Enterococcus* (VRE), linezolid has shown promising activity against drug-resistant TB. Linezolid-containing regimens show better efficacy in MDR/XDR-TB treatment, including faster cavity closure and higher sputum culture conversion rate, which is important for reducing transmission [4,5].

However, the treatment of drug-resistant tuberculosis often requires a long duration of therapy, and prolonged use of linezolid has been associated with a significant risk of adverse drug reactions (ADRs) [6,7,8,9]. The most common ADRs related to linezolid include hematological toxicity, while others such as peripheral neuropathy, gastrointestinal disturbances, and visual impairment have also been reported. Several studies have found that patients treated with linezolid experience a higher incidence of ADRs within two weeks of therapy compared to those not receiving linezolid [9,10,11,12,13,14,15]. Although several reviews have examined linezolid-related ADRs [16,17,18], only a limited number have specifically focused on drug-resistant TB, with most existing reviews evaluating ADRs associated with linezolid in other infections. Therefore, this systematic review aims to examine the incidence, types, and frequency of linezolid-related adverse drug reactions in drug-resistant tuberculosis.

## 2. Materials and Methods

### 2.1. Study Design

**Figure 1:**
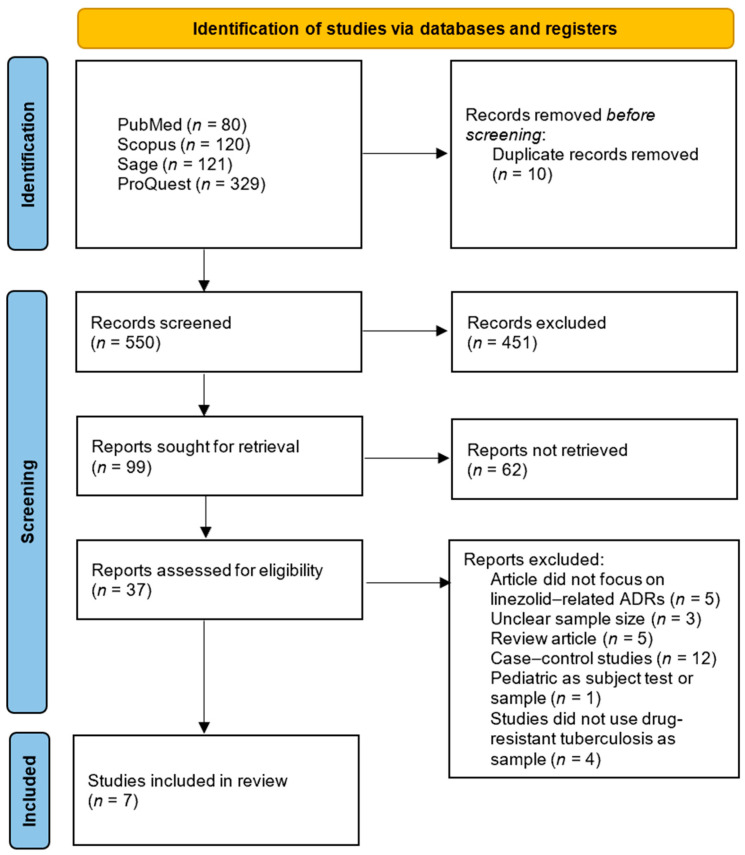
PRISMA 2020 flow diagram.

This systematic review was conducted and reported in accordance with the Preferred Reporting Items for Systematic Reviews and Meta-Analyses (PRISMA) 2020 guideline to ensure methodological transparency and reproducibility [19]. A structured protocol was developed prior to the literature search, specifying the objectives, eligibility criteria, data extraction process, and quality assessment strategy. Although the protocol was not registered in any database, the full methodology is detailed within this manuscript. No amendments were made as no protocol was registered. A PRISMA flow diagram summarizing the study selection process is presented in Figure 1. The complete PRISMA 2020 checklist is provided in the Supplementary Materials.

### 2.2. Literature Source

The relevant studies were identified using four databases, consisting of PubMed, Scopus, ProQuest, and Sage, with keywords arranged using a MeSH database. However, adjustments were made to ensure optimal search results. We proceeded to hand search the article list, reviewed it, and screened it to identify eligible articles. Literature selection was not limited by year. The list of keywords can be seen on Table 1.

### 2.3. Eligibility Criteria and Selection Process

The eligibility criteria included quantitative or qualitative studies discussing linezolid-related ADRs in drug-resistant tuberculosis which were available in full text and written in Indonesian or English. Exclusion criteria included articles that were case reports, reviews, protocols, case series, clinical guidelines, book chapters, or letters to the editor; studies involving subjects who were pediatric, pregnant, or lactating women; and studies that did not use linezolid or did not use linezolid as an individual regimen.

Only articles published up to June 2023 were considered. The selection process was conducted in two stages: (1) initial title and abstract screening and (2) full-text review. In the initial stage, three independent reviewers screened titles and abstracts to determine relevance. Screening was conducted using Mendeley Reference Manager to organize eligible articles and automatically identify duplicates. Disagreements were resolved through discussion, and irrelevant articles were moved to a separate folder. After the initial stage, three independent reviewers conducted a full-text review and discussed any disagreements. The review assessed whether patients had drug-resistant tuberculosis treated with linezolid, whether patients experienced ADRs, the type and timing of ADRs, and subsequent actions taken. No eligible articles were excluded due to reviewer disagreement.

### 2.4. Data Extraction

The primary outcome of this review was the type and frequency of linezolid-related ADRs, such as hematological and neurologic toxicity. Information extracted from eligible studies included the author(s), year of publication, study location, study design, study population, linezolid dose, frequency of ADRs, and time to onset of the three main types of ADRs (hematologic toxicity, peripheral neuropathy, and optic neuropathy), and distribution of other ADRs. ADR markers were tailored to each study. Data extraction was independently conducted by two reviewers using a standardized Excel form and cross-checked by a third reviewer to ensure accuracy. Discrepancies were resolved by discussion. Unclear data were recorded as “Not applicable (N/A)” and “Not reported (NR)” if not included. No assumptions were made regarding missing data. Potential reporting bias was minimized by comprehensive database search and duplicate screening.

### 2.5. Quality Assessment

To evaluate the method quality and risk of bias of the included retrospective cohort or prospective studies, we used the Joanna Briggs Institute (JBI). Three independent reviewers conducted a quality assessment of the studies, with any disagreements resolved through discussion with each other. The results of the JBI appraisal were categorized into high quality (>70%), medium quality (50–70%), and low quality (<50%), which would be considered indicative of high risk of bias.

## 3. Results

### 3.1. Literature Search

The initial search using four databases yielded 650 articles: 80 from PubMed, 120 from Scopus, 121 from Sage, and 329 from ProQuest, as shown in Figure 1. There were 100 duplicate articles, leaving 550 articles to be screened by title and abstract. The title and abstract screening results showed that 99 articles remained, and 451 articles were excluded because the title and abstract did not focus on linezolid, ADRs, and tuberculosis. There were 99 articles used for full-text screening. These 99 articles were screened again by downloading their full-text versions. As a result, 37 articles were successfully retrieved in full-text form, while 62 articles could not be accessed due to access restrictions or errors. From the total of 37 articles, a full-text review was then conducted to identify studies that met the inclusion criteria. A total of seven articles met the inclusion criteria and were eligible for review. A total of 30 articles were excluded because of some reasons, consisting of 5 articles not focusing on linezolid-related ADRs, 3 being unclear about sample size, 5 articles being reviews, 12 articles being case–control studies, 1 article using pediatric test subjects, and 4 articles not using drug-resistant tuberculosis patients as their sample. The JBI checklist was used to evaluate the quality of each article. The results indicated that the scores ranged from 63.6 to 100%, and all seven articles had medium- to high-quality research designs. Table 2 displays the results of the quality appraisal.

### 3.2. Study Characteristics

All seven articles used prospective or retrospective cohorts in their study design. The sample sizes varied from 8 patients (smallest) to 195 patients (largest). On average, research subjects were of productive age, and just two articles used the elderly as research subjects, namely Xu et al. [20] and Padmapriyadarsini et al. [21]. Judging from the type of research subjects, all articles used drug-resistant tuberculosis patients as the research subjects. All of the articles were about linezolid-related ADRs. Evaluation of ADRs was carried out using medical records, follow-up procedures, or both, and also researchers explored the relationship between ADRs and other factors that could be related. From seven studies, just two studies reported about previous history of TB treatment in MDR/XDR-TB patients. Most of the patients included in these studies had a previous history of TB treatment (all patients included [20], and 111 patients of 151 patients included [12]), but there was no more information about patients’ history of treatment of TB.

Judging from the dose of linezolid used, it varied from 300 to 1200 mg [22]. Most of them used 600 mg twice daily or 1200 mg once daily orally. There is one study that used 1200 mg of linezolid intravenously. Linezolid is a type of antibiotic that is widely used in drug-resistant tuberculosis. Several studies state that linezolid has a high risk of ADRs. The seven studies are spread across several regions: South Korea with 2 studies, South Africa with 2 studies, and China, Germany, and India with 1 study each. This could have been influenced by the small number of articles identified. The characteristics of each linezolid-related ADRs of the seven studies can be seen in Table 3 and Table 4.

**Table 2:** Results of quality assessment using JBI appraisal.

	JBI Appraisal Questions and the Value											
Author, Year	1	2	3	4	5	6	7	8	9	10	11	Appraisal Result
Park et al., 2006 [22]	√	√	√	√	×	×	√	×	√	√	√	Included
Xu et al., 2012 [20]	-	-	√	√	√	√	√	√	√	-	√	Included
Migliori et al., 2009 [23]	√	√	√	-	-	√	√	-	×	√	√	Included
Koh et al., 2009 [24]	√	√	√	√	×	×	√	√	√	×	√	Included
Wasserman et al., 2022 [12]	-	-	√	√	√	×	√	√	√	√	√	Included
Imperial et al., 2022 [25]	√	√	√	√	√	√	√	√	√	√	√	Included
Padmapriyadarsini et al., 2023 [18]	√	√	√	√	√	×	√	√	√	√	√	Included

(√): yes; (×): no; (-): not available. Question 1: Were the two groups similar and recruited from the same population? Question 2: Was the exposure measured similarly to assign people to both exposed and unexposed groups? Question 3: Was the exposure measured in a valid and reliable way? Question 4: Were confounding factors identified? Question 5: Were strategies to deal with confounding factors stated? Question 6: Were the groups/participants free of the outcome at the start of the study (or at the moment of exposure)? Question 7: Were the outcomes measured in a valid and reliable way? Question 8: Was the follow-up time reported sufficient to be long enough for outcomes to occur? Question 9: Was follow-up complete, and if not, were the reasons for loss to follow-up described and explored? Question 10: Were strategies to address incomplete follow-up utilized? Question 11: Was appropriate statistical analysis used?

**Table 3:** Characteristics of hematologic toxicity from included studies.

Author, Year	Study Location/Study Design	Sample Size	Dose of Linezolid in Study	Previous History TB (Yes/No/N/A)	Hematologic Toxicity				
					Amount	Time of Occurrence	Linezolid Adjustment	Linezolid Discontinuation	Symptomatic Therapy
Park et al., 2006 [22]	South Korea/Prospective cohort	Total: 8 patients all MDR-TB	2 patients: 600 mg once daily 5 patients: 600 mg twice daily for 2 weeks 1 patient: 600 mg twice daily for 7 weeks	NA	Total: 1 incident Anemia (Hb 9.6 mg/dL): 1 patient (600 mg twice daily)	After 7 weeks	600 mg once daily	-	-
Xu et al., 2012 [20]	China/Retrospective cohort	Total 18 patients. 15 patients with XDR-TB and 3 patients with MDR-TB	1. 1200 mg once daily IV drip infusion at treatment initiation for all patients. 2. 3 patients adjusted for body weight for 900 mg once daily	Yes: All patients	Total: 13 incidents Anemia: 2 incidents (900 mg once daily), 10 incidents (1200 mg once daily) Mild to moderate thrombocytopenia (Platelet 50–109 × 10^9^/L): 3 incidents (1200 mg once daily) Leucopenia (<4.0 × 10^9^/L): 7 incidents (1200 mg once daily)	Anemia: 2 incidents (900 mg once daily) occurred after 19 days 10 incidents (1200 mg once daily) occurred after 80.1 days Mild to moderate thrombocytopenia (Platelet 50–109 × 10^9^/L): 3 incidents (1200 mg once daily) Leucopenia (<4.0 × 10^9^/L): 7 incidents (1200 mg once daily)	Anemia: 2 incidents (900 mg once daily) adjusted to 600 mg once daily 10 incidents (1200 mg once daily): 8 incidents adjusted to 600 mg once daily, 3 incidents adjusted to 900 mg once daily, and 1 incident adjusted to 300 mg once daily Leucopenia: 5 incidents (1200 mg once daily) adjusted to 600 mg once daily 2 incidents adjusted to 900 mg once daily Thrombocytopenia: 2 incidents (1200 mg once daily) adjusted to 600 mg once daily 1 incident adjusted to 900 mg once daily		7 incidents managed by combination of erythropoietin, blood transfusion, folic acid, and iron supplementation.
Migliori et al., 2009 [23]	Germany/Retrospective cohort	Total 195 MDR/XDR-TB. 85 treated with linezolid 110 without linezolid. From 85 with linezolid, 75 patients MDR-TB and 10 patients with XDR-TB.	1. 28 patients: 600 mg once daily 2. 57 patients: 600 mg twice daily	N/A	Total: 30 incidents Anemia: 3 incidents (600 mg once daily), 20 incidents (600 mg twice daily) Thrombocytopenia: 7 incidents (600 mg twice daily)	69 days	Yes	Yes	Yes (5 requiring blood transfusion)
Koh et al., 2009 [24]	South Korea/Retrospective cohort	Total 24 patients all MDR-TB	300 mg once daily: 17 patients 600 mg once daily: 7 patients	N/A	Total: 1 incident Leucopenia: 1 incident (600 mg once daily)	104 days (600 mg once daily)	Adjusted to 300 mg once daily after temporarily discontinuation		
Wasserman et al., 2022 [12]	South Africa/Prospective cohort	Total 151 patients all MDR-TB	600 mg once daily: 148 patients 300 mg once daily: 3 patients	Yes: 111 patients from 151 patients	Total: 74 incidents Anemia: 58 incidents Grade 1: 24 Grade 2: 14 Grade 3: 13 Grade 4: 6 Thrombocytopenia: 10 incidents Grade 1: 8 Grade 2: 2 Leukopenia: 6 incidents Grade 1: 5 Grade 3: 1	Anemia: 77 days	Yes	Yes	
Imperial et al., 2022 [25]	South Africa/Retrospective cohort	Total 109 patients all MDR-TB	600 mg twice daily or 1200 mg once daily	N/A	Total: 44 incidents Anemia: 38 incidents Thrombocytopenia: 6 incidents	56 days	Yes	Yes	
Padmapriyadarsini et al., 2023 [18]	India/Prospective cohort	Total 165 patients all MDR-TB	600 mg once daily	N/A	Anemia: 78 incidents	56 days	Yes, 600 mg to 300 mg once daily	-	Yes, with hematinic support. One patient received blood transfusion.

N/A: Not applicable.

**Table 4:** Characteristics of polyneuropathy toxicity from included studies.

Author, Year	Study Location/Study Design	Sample Size	Dose of Linezolid in Study	Previous History TB (Yes/No/N/A)	Polyneuropathy Toxicity				
					Amount	Time of Occurrence	Linezolid Adjustment	Linezolid Discontinuation	Symptomatic Therapy
Park et al., 2006 [22]	South Korea/Prospective cohort	Total: 8 patients all MDR-TB	1. 2 patients: 600 mg once daily 2. 5 patients: 600 mg twice daily for 2 weeks 3. 1 patient: 600 mg twice daily for 7 weeks	N/A	Peripheral neuropathy: Total: 4 incidents (600 mg twice daily) Optic neuropathy: 2 incidents (600 mg twice daily)	Peripheral neuropathy: 120–330 days Optic neuropathy: 240–270 days	Peripheral neuropathy: 600 mg once daily then symptomatic therapy (Amitriptyline, gabapentin, vitamin B6)	Optic neuropathy: Linezolid discontinued	Amitriptyline, gabapentin, vitamin B6
Xu et al., 2012 [20]	China/Retrospective cohort	Total 18 patients. 15 patients with XDR-TB and 3 patients with MDR-TB	1. 1200 mg once daily IV drip infusion at treatment initiation for all patients. 2. 3 patients adjusted for body weight for 900 mg once daily	Yes: All patients	Peripheral neuropathy: Total: 11 incidents 9 incidents (1200 mg once daily) 1 incident (900 mg once daily) Optic neuropathy: Total: 3 incidents 1 incident (900 mg once daily) 2 incidents (1200 mg once daily)	Peripheral neuropathy: 9 incidents (1200 mg once daily) occurred after 90.8 days 1 incident (900 mg once daily) occurred after 7 days Optic neuropathy: 1 incident (900 mg once daily) occurred after 7 days 2 incidents (1200 mg once daily) occurred after 47.5 days	Peripheral neuropathy: 7 incidents adjusted to 600 mg once daily 1 incident adjusted to 900 mg once daily 1 incident adjusted to 300 mg once daily Symptomatic therapy: Vitamin B6, and B12 Optic neuropathy: 600 mg once daily. 1 incident stop cause of worsening. Symptomatic therapy: Vitamin B12	Optic neuropathy: After linezolid adjusted to 600 mg once daily and symptomatic therapy, 1 incident discontinued of linezolid cause of worsening.	
Migliori et al., 2009 [23]	Germany/Retrospective cohort	Total 195 MDR/XDR-TB. 85 treated with linezolid 110 without linezolid. From 85 with linezolid, 75 patients MDR-TB and 10 patients with XDR-TB.	1. 28 patients: 600 mg once daily 2. 57 patients: 600 mg twice daily	N/A	Total: 3 incidents Polyneuropathy: 1 incident (600 mg once daily), 2 incidents (600 mg twice daily)	69 days	N/A	Discontinued on some cases	
Koh et al., 2009 [24]	South Korea/Retrospective cohort	Total 24 patients all MDR-TB	300 mg once daily: 17 patients 600 mg once daily: 7 patients	N/A	Total: 8 incidents Peripheral neuropathy: 4 incidents (300 mg once daily) 4 incidents (600 mg once daily)	289 days (300 mg once daily) 104 days (600 mg once daily)	4 incidents (600 mg once daily) adjusted to 300 mg once daily	1 incident (300 mg once daily) discontinued	
Wasserman et al., 2022 [12]	South Africa/Prospective cohort	Total 151 patients all MDR-TB	600 mg once daily: 148 patients 300 mg once daily: 3 patients	Yes: 111 patients from 151 patients	Total: 54 incidents Peripheral neuropathy: 37 incidents Grade 1: 32 incidents Grade 2: 4 incidents Grade 3: 0 Grade 4: 1 incident Optic neuropathy: 17 incidents	Peripheral neuropathy: 70 days Optic neuropathy: 70 days	Yes	Yes	
Imperial et al., 2022 [25]	South Africa/Retrospective cohort	Total 109 patients all MDR-TB	600 mg twice daily or 1200 mg once daily	N/A	Peripheral neuropathy: 80 incidents	98 days	Yes	Yes	
Padmapriyadarsini et al., 2023 [18]	India/Prospective cohort	Total 165 patients all MDR-TB	600 mg once daily	N/A	Total: 71 incidents Peripheral neuropathy: 69 incidents Optic Neuropathy: 2 incidents	Peripheral neuropathy: 112 days Optic Neuropathy: 60 days	-	-	Yes

N/A: Not applicable.

### 3.3. Profile of Linezolid-Related Adverse Events

Based on the pooled estimate from the included studies, the proportion of anemia among the population was 0.32 (95% CI: 0.11–0.53). This suggests that approximately one-third of individuals in the analyzed cohort experienced anemia, although the confidence interval indicates notable uncertainty in the precise magnitude. The individual study estimates varied widely, ranging from 0.00 to 0.67, demonstrating substantial variability across study populations, methodologies, and geographic or clinical contexts. Despite this variation, most point estimates fell within the mild-to-moderate range of anemia prevalence, highlighting the relevance of anemia as a potential clinical concern within this setting.

**Figure 2:**
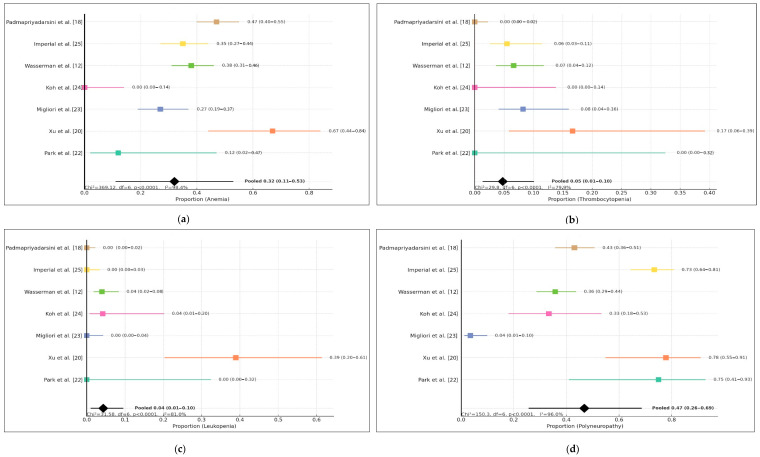
Forest plot of (**a**) anemia, (**b**) thrombocytopenia, (**c**) leukopenia, (**d**) polyneuropathy.

The heterogeneity assessment further supports the variability among studies, with a test statistic of Chi^2^ = 369.12, df = 6, *p* < 0.0001, indicating that the observed differences across studies were unlikely to be due to chance alone. The high inconsistency value (I^2^ = 98.4%) confirms considerable heterogeneity, which may reflect differences in study design, participant characteristics, diagnostic criteria, or treatment exposure. Therefore, while the pooled estimate provides an overall summary, interpretation should consider the wide variability and potential contextual modifiers influencing anemia prevalence across studies.This data can be seen in Figure 2.

The pooled analysis of thrombocytopenia events across seven included studies demonstrated an overall proportion of 5% (95% CI: 1–10%), indicating that thrombocytopenia occurred less frequently than other hematologic adverse reactions reported during linezolid use. However, the wide confidence interval and the presence of studies reporting zero events suggest variability in reporting and potential under-detection in smaller cohorts. Individual study estimates varied, with Xu et al. [20], Migliori et al. [23], Wasserman et al. [12], and Imperial et al. [25] reporting measurable cases, while Park et al. [22], Koh et al. [24], and Padmapriyadarsini et al. [18] reported none.

Significant between-study heterogeneity was observed (Chi^2^ = 29.80, df = 6, *p* < 0.0001; I^2^ = 79.9%), suggesting that differences in patient characteristics, monitoring frequency, comorbidities, or treatment regimens may influence the detection and incidence of thrombocytopenia. Despite the heterogeneity, the overall low pooled proportion suggests that thrombocytopenia remains a relatively uncommon adverse hematologic event associated with linezolid therapy. Continued pharmacovigilance is warranted, particularly in settings with limited laboratory monitoring or in populations with pre-existing hematologic vulnerabilities.

The pooled analysis of leukopenia demonstrated a relatively low overall proportion, with a combined estimate of 0.04 (95% CI 0.01–0.10) based on seven included studies. Although leukopenia cases were reported across all studies, the majority documented only a small percentage of affected participants, consistent with earlier evidence suggesting that hematologic suppression from the evaluated regimen occurs less frequently compared with other adverse events such as anemia or thrombocytopenia. Despite the low pooled proportion, confidence intervals across individual studies varied widely, reflecting differing population characteristics, study designs, and treatment durations.

Statistical assessment revealed substantial heterogeneity (I^2^ = 81.0%, χ^2^ = 31.58, df = 6, *p* < 0.0001), indicating that variability was unlikely to be due to chance alone. This suggests that differences among study populations, diagnostic thresholds, or treatment monitoring protocols may have influenced the observed incidence. Nevertheless, the overall findings support that leukopenia remains a relatively infrequent hematologic complication in the included clinical contexts, reinforcing the safety profile observed in the manuscript and accompanying analyses.

The pooled proportion of polyneuropathy was notably higher compared with hematologic adverse events, with a combined estimate of 0.29 (95% CI 0.19–0.41) across the seven included studies. Most studies reported a meaningful number of polyneuropathy cases, indicating that this adverse event represents one of the more frequent treatment-related toxicities in this population. The proportion varied considerably across studies, ranging from relatively low rates in earlier cohorts to substantially higher proportions in more recent publications. This pattern may reflect improved detection, differences in treatment duration, cumulative exposure, or population-specific risk factors.

Heterogeneity across studies was substantial (I^2^ = 89.3%, χ^2^ = 56.23, df = 6, *p* < 0.0001), demonstrating that the observed variability was unlikely to be due to random variation. Contributing factors may include differences in diagnostic criteria, baseline nutritional status, or coexisting conditions such as diabetes or HIV infection, which are known to predispose patients to neurotoxicity. Despite this variability, the pooled estimate suggests that polyneuropathy represents a significant clinical concern and requires close monitoring during treatment. These findings reinforce the importance of early recognition and supportive management strategies to minimize functional impairment and improve treatment tolerability.

### 3.4. Time-Dependent Hematologic and Polyneuropathy Events of Linezolid

Hematologic toxicity associated with prolonged linezolid therapy consistently appeared in the early phase of treatment. Anemia and thrombocytopenia were most frequently observed between weeks 8 and 16, regardless of dosing strategy (300–600 mg once daily or 600 mg twice daily followed by once daily). This pattern reflects cumulative myelosuppression related to prolonged drug exposure rather than peak concentrations. The consistency of this onset window across studies indicates a predictable hematologic risk, supporting the need for routine laboratory monitoring starting within the first two months of therapy.

**Figure 3:**
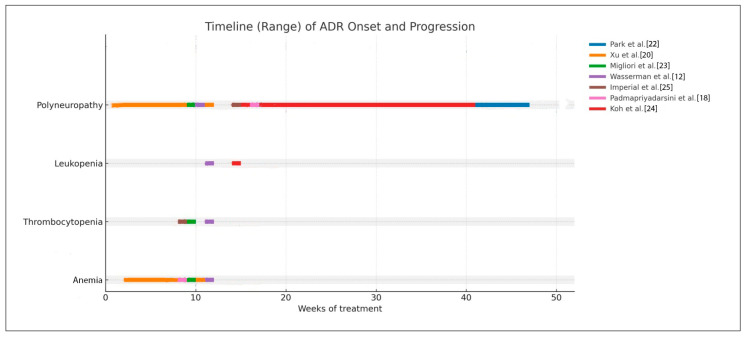
Timeline chart of the temporal relationship between dose or duration and ADR occurrence.

In contrast, peripheral neuropathy showed a delayed onset, typically occurring after week 16, with some earlier cases in patients receiving higher initial doses. This pattern supports a cumulative mitochondrial toxicity mechanism rather than an acute pharmacologic effect. These findings emphasize that monitoring strategies should be adjusted based on ADR type: early and regular hematologic monitoring during initial treatment and intensified neurotoxicity surveillance after three months or during prolonged regimens. This timeline-based analysis strengthens the rationale for dose optimization and tapering strategies to reduce cumulative toxicity and maintain tolerability in long-term multidrug-resistant tuberculosis treatment. In addition to that, to manage linezolid-related ADRs, several strategies are needed for ADR definition, treatment, and prevention through clinical assessment. This strategy can be seen in Table 5, and Figure 3.

## 4. Discussion

### 4.1. Hematologic Toxicity

Hematologic toxicity, including anemia, thrombocytopenia, and leukopenia, represents one of the most clinically significant ADRs associated with linezolid [26,27]. Wasserman et al. [12] observed 58 cases of anemia among 151 MDR-TB patients receiving linezolid, accompanied by 10 incidents of thrombocytopenia and 6 incidents of leukopenia after a median of 77 days of therapy. Similarly, Imperial et al. [25] reported 44 incidents of hematologic toxicity events among 109 patients, with 38 incidents of anemia and 6 incidents of thrombocytopenia, typically occurring around 56 days after treatment initiation. The earliest onset of hematologic toxicity was reported at approximately seven weeks of linezolid use [22], while the latest onset occurred after 104 days [24]. These findings indicate that hematologic abnormalities generally emerge during the early months of therapy. This result can be seen on Table 3.

With regard to linezolid dosing, hematologic toxicity was most frequently reported at doses of 600 mg once daily, 600 mg twice daily, and 1200 mg once daily. The highest number of anemia cases occurred with the 600 mg once-daily regimen, with 78 incidents among 165 MDR-TB patients [18], followed by 58 incidents among 151 patients in another study [12]. For the 600 mg twice-daily regimen, two studies reported 38 anemia incidents among 109 patients [25] and 20 incidents among 195 patients [23], respectively. Meanwhile, at the 1200 mg once-daily dose, one study documented 10 incidents of anemia [20].

Thrombocytopenia is the second most commonly reported hematologic toxicity, with three studies documenting its association with linezolid use. One study showed that there are 10 incidents of thrombocytopenia from 600 mg once daily [12], and 2 studies reported thrombocytopenia incidents from 600 mg twice daily [23,25]. Leukopenia, although less frequent, has also been reported in patients receiving linezolid at 600 mg once daily (2 studies: 1 incident [24] and 6 incidents [12], and 1 study at 1200 mg once daily (7 incidents) [20]. Across studies, the majority of anemia, thrombocytopenia, and leukopenia occurred at linezolid doses of 600 mg once daily, 600 mg twice daily, or 1200 mg once daily. These data suggest that toxicity is influenced not only by the daily dose but also by cumulative exposure. The dose–toxicity relationship is further supported by findings from Migliori et al. [23], who reported a significant difference in hematologic toxicity between 600 mg once daily and 1200 mg once daily (*p* = 0.0004). However, multiple studies also observed hematologic toxicity at the 600 mg once-daily dose, indicating that adverse effects are both dose-dependent and duration-dependent. Given that linezolid is typically administered for 18–24 months in MDR-TB regimens, prolonged exposure likely contributes substantially to the risk of hematologic toxicity.

According to several studies, hematological toxicity can occur at the beginning of therapy with linezolid. As is known, linezolid is used as one of the drugs in a drug-resistant tuberculosis therapy regimen for 18–24 months. Linezolid-related ADRs are dose-dependent and duration-dependent. Long-term use of linezolid can increase the risk of ADRs such as hematological toxicity [7,26,27,28,29,30,31,32,33,34,35,36]. Linezolid can cause anemia due to mitochondrial dysfunction. Linezolid can induce Pure Red Cell Aplasia (PRCA) pro-toxicity and inhibit mitochondrial respiration by breaking down mitochondrial protein synthesis and inhibiting protein synthesis. The highest incidence of ADRs is related to spinal cord compression. This incident occurs on average within 10–14 days of using linezolid. Two mechanisms comprise it: the first is spinal cord suppression, where linezolid can increase myosin light chain 2 phosphorylation, followed by repression of platelet release from mature megakaryocytes. The second mechanism is increased platelet destruction by the immune system. Linezolid can suppress the proliferation and activity of cellular metabolites and interfere with mitochondrial function [11,33,36,37,38,39,40]. Several study results state that anemia is reversible after the use of linezolid is stopped, but this depends on the severity of the ADR and the patient’s clinical condition.

Management of hematologic toxicity typically involves dose reduction, temporary interruption, or discontinuation of linezolid, accompanied by supportive treatment such as folic acid supplementation. In severe cases, patients may require blood transfusion. Several studies observed improvement in anemia following dose reduction to 600 mg once daily, highlighting the clinical importance of individualized dosing adjustments. Collectively, the evidence indicates that hematologic toxicity related to linezolid is multifactorial—driven by dose, duration, and individual susceptibility. Early recognition of ADRs and timely dose modification are essential to maintaining therapeutic efficacy while minimizing harm. These findings underscore the need for routine hematologic monitoring, particularly during the early months of therapy and in patients receiving higher cumulative exposure to linezolid.

### 4.2. Peripheral Neuropathy

Neurotoxicity is also a frequently reported ADRs [21,31,41,42]. Wasserman et al. [12] showed that of 151 patients, 54 incidents involved peripheral neuropathy after 70 days of use of linezolid [12]. Imperial et al. [25] also showed peripheral neuropathy in as many as 80 incidents at 98 days of linezolid use. Likewise, in Park et al. [22], there were four incidents from a total of eight patients included in the study, with time of occurrence at 120–330 days. The incidence of peripheral neuropathy was not related to the dose but instead to the duration of linezolid [13,43]. Koh et al. [24] showed that of 17 patients who were given linezolid at 300 mg once daily and 7 patients at 600 mg once daily for 289 days, peripheral neuropathy occurred at an amount of 8 incidents. This adverse event occurred earlier for those taking 600 mg of linezolid once daily. The five patients received doses reduced from 600 mg once daily to 300 mg once daily, and the peripheral neuropathy did not improve. The earliest instance of peripheral neuropathy occurred after 7 days of linezolid use and the longest at 120–330 days after linezolid use.

In the study of Xu et al. [20], neurotoxicity was also observed. Neurotoxicity occurred 11 times, of which 10 incidents occurred from use of 600 mg of linezolid twice daily, and 1 incident from 900 mg once daily. In contrast to this result, Migliori et al. [23] showed a lower incidence rate of peripheral neuropathy (3 incidents from 85 patients using linezolid). Padmapriyadarsini et al. [18] also reported that there were 69 patients who experienced peripheral neuropathy after 112 days of therapy.

Peripheral neuropathy caused by linezolid often appears with symptoms such as pain in the legs, numbness, tingling, and pain when touching clothing or cold water. The mechanism of peripheral neuropathy is inhibition of Schwann cell proliferation. Linezolid can inhibit autophagy flux in Schwann nerve tissue which results in loss of the myelin sheath or growth inhibition [7,15,16,43,44,45,46]. Peripheral neuropathy often occurs in patients with moderate to high severity. Peripheral neuropathy symptoms are often unknown to individuals. New patients begin to complain and report these symptoms as soon as they worsen. Peripheral neuropathy is hence frequently discovered too late. Reducing the dose and discontinuing linezolid are often interventions that are performed to overcome these ADRs. In contrast to anemia, peripheral neuropathy is sometimes irreversible or takes a long time to return to normal. The supportive treatments that are usually used to manage peripheral neuropathy symptoms are amitriptyline, gabapentin, and pyridoxin.

### 4.3. Optic Neuropathy

Apart from hematologic toxicity and peripheral neuropathy, optic neuropathy is also one linezolid-related ADR. Optic neuropathy can occur with or without symptoms. Usually, the symptoms are visual disturbances, blurred eyes, reduced visual acuity, loss of color vision, and even loss of vision [16,43,45,47]. Of the seven studies, just five studies reported optic neuropathy. Wasserman et al. [12] stated that optic neuropathy occurred in total 17 incidents after 70 days of linezolid use. Park et al. [22] showed, that apart from anemia and peripheral neuropathy, optic neuropathy occurred two times at 240–270 days after linezolid use. After linezolid was stopped, the optic neuritis improved after 2–3 months in both patients.

Three incidents of optic neuropathy were reported by Xu et al. [20], involving one patient using 900 mg of linezolid once daily and two patients using 1200 mg once daily. This event occurred at varying time points (one incident at 900 mg once daily at 7 days, and two incidents at 1200 mg once daily at 47.5 days after linezolid use). After linezolid was stopped, vision improved again within 2–3 weeks. The same incident was also reported by Padmapriyadarsini et al. [18] where out of 165 patients with MDR-TB, there occurred two incidents of optic neuropathy. The incidence of optic neuropathy tends to be lower than those of hematological toxicity and peripheral neuropathy. Optic neuritis will improve when linezolid is stopped, but it takes a long time [45,48,49,50,51,52,53].

### 4.4. Another Type of Linezolid-Related ADRs

Hyperlactatemia is a linezolid-related ADR that is also associated with mitochondrial disorders. Only one article reported this incident. There is a hypothesis that the incidence of hyperlactatemia is related to genetic susceptibility to linezolid toxicity through linezolid binding to mitochondria. Hyperlactatemia is also associated with lactic acidosis, so linezolid has a risk of causing lactic acidosis. Lactic acidosis is associated with poor clinical outcomes, including high lactate which can cause organ dysfunction and death. The underlying mechanism is the inhibition of mitochondrial oxidative phosphorylation which leads to hypoxia. In addition, a mitochondrial DNA polymorphism (A2706G) is also associated with lactic acidosis [54].

Other linezolid-related ADRs include gastrointestinal disorders and rash. Gastrointestinal disorders can occur in 58,9% of linezolid users at doses of 600 mg or more [4,55]. Xu et al. [20] reported on gastrointestinal disorders, where it happened as much as 15 times for nausea and diarrhea, which included 13 incidents at 1200 mg once daily and 2 incidents at 900 mg once daily. These gastrointestinal disorders were treated with anti-emetics or anti-diarrheal medication. Rash also occurred in 10 patients with linezolid use at 600 mg twice daily, and 6 of them received anti-histamine therapy. Migliori et al. [23] also reported that there were four incidents of nausea and vomiting (one incident from 600 mg of linezolid once daily, and three incidents from 600 mg twice daily). These adverse events occurred after 69 days of linezolid use.

From several studies, it can be seen that linezolid-related ADRs consist of hematologic toxicity, neurotoxicity, gastrointestinal disorder, and hyperlactatemia. The risk of linezolid-related ADRs is based on dose and the duration of linezolid use. Some ADRs can be resolved by reducing the dose or discontinuation of linezolid, while some conditions are irreversible, such as peripheral neuropathy and optic neuropathy. Several studies also explain that one of the factors related to ADRs is linezolid concentration in the blood. A high concentration exceeding the therapeutic range of linezolid is closely related to the occurrence of linezolid-related ADRs. Thus, monitoring linezolid in the blood, especially in MDR/XDR-TB, is important to prevent ADRs [56,57,58].

Linezolid is a type of antibiotic with good effectiveness against MDR-TB. However, the high number of reports of linezolid-related ADRs is also a concern, especially in relation to the dose regimen. Several studies also monitored linezolid in blood and showed its correlation with ADRs. Many factors are correlated to ADRs such as comorbidities, dose regimen, duration of use of linezolid, and use of other drugs. One step to maximize the therapeutic effect and minimize the ADR risk can be to perform therapeutic drug monitoring in the context of individualizing the linezolid dose.

### 4.5. Clinical Implication, Practical ADR Management, and Monitoring Risk

The consolidated timeline analysis revealed distinct temporal patterns in ADRs associated with prolonged linezolid therapy. Hematologic toxicities, particularly anemia and thrombocytopenia, consistently emerged during the earlier phase of treatment, with most cases occurring between weeks 8 and 16 across included studies, irrespective of dosing strategy (300–600 mg once daily or 600 mg twice daily transitioned to once daily). This temporal clustering aligns with the known mechanism of linezolid-induced myelosuppression, which is primarily driven by cumulative drug exposure rather than peak plasma concentration. The reproducibility of this onset window across multiple cohorts suggests a predictable hematologic risk profile and supports the implementation of routine laboratory monitoring beginning within the first two months of therapy.

In contrast, peripheral neuropathy demonstrated a delayed and broader onset distribution, typically arising after extended treatment durations. Most cases were reported after week 16, although earlier occurrences were observed in regimens incorporating higher initial doses (e.g., 600 mg twice daily). The pattern observed across the included literature indicates that neuropathy is predominantly cumulative in nature and reflects mitochondrial toxicity rather than immediate pharmacologic effect. These findings are consistent with prior mechanistic understanding and reinforce that neuropathy risk increases substantially when treatment extends beyond standard durations used for non-tuberculosis indications.

Collectively, these observations underscore the importance of tailoring monitoring strategies according to ADR onset profiles. Hematologic parameters should be assessed early and at regular intervals during the initial phases of therapy, while surveillance for neurotoxicity may be more appropriately intensified after three months of treatment or during dose-prolonged regimens. The unified timeline format used in this analysis synthesizes heterogeneous clinical data and provides a comparative framework illustrating ADR risk over time. These findings support the rationale for dose optimization strategies—such as reduced maintenance dosing or structured tapering—particularly in prolonged multidrug-resistant tuberculosis regimens, where limiting cumulative toxicity is critical to maintaining treatment tolerability and adherence.

### 4.6. Strengths and Limitations

This review has several notable strengths that enhance its scientific and clinical relevance. First, the literature search was comprehensive and systematically conducted across four major databases (PubMed, Scopus, ProQuest, and SAGE) without year restrictions, minimizing the risk of missing relevant evidence. Second, this review applied strict eligibility criteria focusing on patients with drug-resistant tuberculosis receiving linezolid-containing regimens, thereby improving the clinical applicability of the findings. Third, the review provides a structured synthesis of the most clinically linezolid-related adverse events: hematological and neurological toxicities. This review also details the adverse event incidence, time of occurrence, and dose duration of linezolid, which enables interpretation of dose- and duration-related toxicity profiles. Additionally, the use of the Joanna Briggs Institute (JBI) critical appraisal tool strengthened methodological rigor by ensuring formal assessment of study quality and risk of bias. Finally, by highlighting the association between dose duration of linezolid and emerging linezolid-related adverse drug reactions, this review offers valuable insights for dose optimization, monitoring strategies, and future research on linezolid safety.

This review has several limitations that should be acknowledged. First, the evidence base is small, with only seven eligible studies, and these studies were heterogeneous in terms of study design, sample size, dosing regimens, follow-up duration, and the parameters used to define adverse drug reactions. This heterogeneity limits the comparability of findings and contributes to uncertainty in the overall conclusions. Second, most included studies were observational cohort designs, and the absence of randomized controlled trials reduces the strength of causal interpretations regarding linezolid-associated adverse events. Third, several studies did not fully report important variables such as the time of occurrence of adverse events, patient characteristics, and adverse event treatment in detail, and other factors which may affect the accuracy of pooled interpretation. Finally, more than half of the potentially relevant full-text articles could not be accessed during screening, introducing a risk of selection bias.

Though this review only included seven eligible articles, the cohort design provides a realistic picture of the high incidence of ADRs due to linezolid use, which is assumed to be influenced by the dose and duration of linezolid administration, so that this review can be a source of information for administering linezolid at the effective dose with minimal side effects and the necessary monitoring aspects. This result can also serve as a basis for determining new treatment regimens for drug-resistant tuberculosis patients, particularly regarding the use of linezolid as one of the therapies. A further implication is to monitor risk by TDM; TDM becomes especially valuable. By measuring plasma levels of linezolid, clinicians may be able to strike a better balance between efficacy and safety [59,60]. This may help physicians reduce the dose without reducing efficacy. The recommended therapeutic range of linezolid is 2 to 7 mg/L for minimum levels, while maximum levels of 7 to 22.1 mg/L are considered the safe upper limit of linezolid’s therapeutic index [9,61]. TDM can be implemented by full sampling time of linezolid pharmacokinetics or sparse pharmacokinetics. There are alternative approaches to start the TDM such as limited sampling strategies (LSS), which have emerged as practical and feasible methods to estimate drug exposure with fewer blood draws and simpler analytical demands [62,63,64]. High levels of linezolid in plasma or serum can affect the efficacy and ADRs of the drug [29]. There is a need to measure drug levels or identify the profile of linezolid levels in the blood and correlate it with clinical conditions that can help identify adverse events.

Further research can be initiated based on this review, which can serve as an initial basis for future research using other experimental or observational research designs to re-evaluate the occurrence of ADRs related to linezolid in drug-resistant tuberculosis patients, particularly at the 600 mg linezolid dose in Indonesia, and research on therapeutic drug monitoring in patients using linezolid at doses of 600 mg or 1200 mg, especially in patients using it long-term.

## 5. Conclusions

Linezolid is used in dose regimens ranging from 300 to 1200 mg, and the use could be accompanied by various ADRs. The most prevalent linezolid-related ADR is hematological toxicity, followed by peripheral neuropathy and optic neuritis. Gastrointestinal disorder, hyperlactatemia, and rash are the least prevalent ADRs. Intensive monitoring of linezolid or TDM in drug-resistant tuberculosis patients based on the drug’s pharmacokinetic profile in the blood or other biological fluids is essential to minimize ADRs incidence.

## Figures and Tables

**Table 1:** Search queries of systematic review.

Database	Search Query	Hits
PubMed	(((tbc OR tuberculosis OR tuberculosis OR “mycobacterium tuberculosis infection” OR “mycobacterium tuberculosis” OR “mycobacterium tuberculosis infections”) AND (“Multidrug-Resistant” OR “Multidrug Resistant” OR MDR OR “Multi-Drug Resistant” OR “Multi Drug Resistant” OR “Drug-Resistant” OR “Drug Resistant”)) AND (Linezolid OR Linezolide OR Zyvox OR Oxazolidinones)) AND (“Drug Related Side Effects and Adverse Reactions” OR “Drug-Related Side Effects and Adverse Reaction” OR “Drug Related Side Effects and Adverse Reaction” OR “Drug Side Effects” OR “Drug Side Effect” OR “effects drug side” OR “Side effect drug” OR “Side effects drug” OR “Adverse Drug Reaction” OR “Adverse Drug Reactions” OR “Drug reaction adverse” OR “Drug reactions adverse” OR “Reactions adverse drug” OR “Adverse drug event” OR “adverse drug events” OR “Drug event adverse” OR “drug events adverse” OR “side effects of drugs” OR “Drug toxicity” OR “toxicity drug” OR “Drug toxicities” OR “toxicities drug”)	80
Scopus	(TITLE-ABS-KEY (tbc OR tuberculosis OR tuberculoses OR “mycobacterium tuberculosis infection” OR “mycobacterium tuberculosis” OR “mycobacterium tuberculosis infections”) AND TITLE-ABS-KEY (“multidrug-resistant” OR “multidrug resistant” OR mdr OR “multi-drug resistant” OR “multi drug resistant” OR “drug-resistant” OR “drug resistant”) AND TITLE-ABS-KEY (linezolid OR linezolide OR zyvox OR oxazolidinones) AND TITLE-ABS-KEY (“drug related side effects and adverse reactions” OR “drug-related side effects and adverse reaction” OR “drug related side effects and adverse reaction” OR “drug side effects” OR “drug side effect” OR “effects drug side” OR “side effect drug” OR “side effects drug” OR “adverse drug reaction” OR “adverse drug reactions” OR “drug reaction adverse” OR “drug reactions adverse” OR “reactions adverse drug” OR “adverse drug event” OR “adverse drug events” OR “drug event adverse” OR “drug events adverse” OR “side effects of drugs” OR “drug toxicity” OR “toxicity drug” OR “drug toxicities” OR “toxicities drug”))	120
Sage	‘tbc OR tuberculosis OR tuberculoses OR “mycobacterium tuberculosis infection” OR “mycobacterium tuberculosis” OR “mycobacterium tuberculosis infections” AND “Multidrug-Resistant” OR “Multidrug Resistant” OR MDR OR “Multi-Drug Resistant” OR “Multi Drug Resistant” OR “Drug-Resistant” OR “Drug Resistant” AND Linezolid OR Linezolide OR Zyvox OR Oxazolidinones AND “Drug Related Side Effects and Adverse Reactions” OR “Drug-Related Side Effects and Adverse Reaction” OR “Drug Related Side Effects and Adverse Reaction” OR “Drug Side Effects” OR “Drug Side Effect” OR “effects drug side” OR “Side effect drug” OR “Side effects drug” OR “Adverse Drug Reaction” OR “Adverse Drug Reactions” OR “Drug reaction adverse” OR “Drug reactions adverse” OR “Reactions adverse drug” OR “Adverse drug event” OR “adverse drug events” OR “Drug event adverse” OR “drug events adverse” OR “side effects of drugs” OR “Drug toxicity” OR “toxicity drug” OR “Drug toxicities” OR “toxicities drug”’	121
ProQuest	(tbc OR tuberculosis OR tuberculosis OR “mycobacterium tuberculosis infection” OR “mycobacterium tuberculosis” OR “mycobacterium tuberculosis infections”) AND (“Multidrug-Resistant” OR “Multidrug Resistant” OR MDR OR “Multi-Drug Resistant” OR “Multi Drug Resistant” OR “Drug-Resistant” OR “Drug Resistant”) AND (Linezolid OR Linezolide OR Zyvox OR Oxazolidinones) AND (“Drug Related Side Effects and Adverse Reactions” OR “Drug-Related Side Effects and Adverse Reaction” OR “Drug Related Side Effects and Adverse Reaction” OR “Drug Side Effects” OR “Drug Side Effect” OR “effects drug side” OR “Side effect drug” OR “Side effects drug” OR “Adverse Drug Reaction” OR “Adverse Drug Reactions” OR “Drug reaction adverse” OR “Drug reactions adverse” OR “Reactions adverse drug” OR “Adverse drug event” OR “adverse drug events” OR “Drug event adverse” OR “drug events adverse” OR “side effects of drugs” OR “Drug toxicity” OR “toxicity drug” OR “Drug toxicities” OR “toxicities drug”)	329

**Table 5:** Linezolid monitoring protocols for early detection, management, and prevention of adverse events.

Domain	Parameters	Frequency	Recommended Actions	Prevention
Hematologic	Complete Blood Count (CBC): hemoglobin, platelets, leukocytes.	Weekly CBC for the first 8 weeks, then every 2 weeks until month 6, then monthly. Monitoring points: baseline, week 2, end of treatment, and follow-up at 6 and 12 months post-treatment.	1. If Hb 10–13 g/dL or platelets 50,000–109,000/µL: provide supportive care or hematinic therapy (e.g., folic acid). 2. If Hb < 10 g/dL or platelets < 100,000/µL: reduce dose to 300 mg once daily. 3. If Hb < 8 g/dL or symptomatic anemia: give blood transfusion and consider immediate discontinuation of linezolid. 4. If linezolid must be discontinued due to adverse reactions, it can be stopped after 9 weeks of therapy. Avoid discontinuation before week 9 unless clinically necessary.	1. Avoid dosing 1200 mg once daily or 600 mg twice daily. 2. For high-risk patients (renal impairment, baseline cytopenia, age > 60 years), consider a lower regimen of 600 mg once daily or 300 mg once daily. 3. Consider therapeutic TDM. Maintain trough concentration < 2 mg/L.
Peripheral neuropathy	Individual assessment using Modified Brief Peripheral Neuropathy Scale (BPNS), Toronto Clinical Neuropathy Score, and symptom screening (paresthesia, numbness, vibration sense, reflexes).	At treatment initiation and monthly during therapy. Monitoring points: baseline, week 2, end of treatment, and follow-up at 6 and 12 months post-treatment.	1. Mild symptoms: continue linezolid and add symptomatic therapy (e.g., vitamin B6, gabapentin). 2. Moderate to severe symptoms: consult a clinician and consider dose adjustment to 300 mg once daily or discontinuation if symptoms progress.	1. Avoid 1200 mg once daily. 2. Early vitamin B6 supplementation may reduce neuropathy progression. 3. Consider TDM. Maintain trough concentration < 2 mg/L.
Optic neuropathy	Snellen chart and Ishihara test.	At treatment initiation and monthly during therapy. Monitoring points: baseline, week 2, end of treatment, and follow-up at 6 and 12 months post-treatment.	1. If symptoms occur (decreased vision, reduced visual acuity, color vision loss): discontinue linezolid immediately. 2. Consider clinician consultation.	1. Consider prednisone 1 mg/kg/day during therapy. 2. Consider TDM. Maintain trough concentration < 2 mg/L.

## Data Availability

The original contributions presented in this study are included in the article/Supplementary Material. Further inquiries can be directed to the corresponding author.

## Supplementary Materials

The following supporting information can be downloaded at: https://www.mdpi.com/article/10.3390/medsci14010003/s1.

## Abbreviations

The following abbreviations are used in this manuscript:

NR	Not reported
N/A	Not applicable
MDR-TB	Multidrug-resistant tuberculosis
XDR-TB	Extensively drug-resistant tuberculosis
ADRs	Adverse drug reactions
JBI	Joanna Briggs Institute
PRISMA	Preferred Reporting Items for Systematic Reviews and Meta-Analyses
MeSH	Medical Subject Headings
RCT	Randomized controlled trials
DNA	Deoxyribonucleic acid
